# Electrically Charged Lipid Nanoparticles as Intracanal Antimicrobial Delivery Systems: A Narrative Review of Preclinical Evidence for Biofilm Control

**DOI:** 10.3390/dj14030171

**Published:** 2026-03-16

**Authors:** Flamur Aliu, Donika Bajrami-Shabani, Javier Flores Fraile, Agron Meto, Cosimo Galletti, Luca Fiorillo, Aida Meto

**Affiliations:** 1 Private Dental Clinic “I Dent”, 10000 Prishtina, Kosovo; flamuraliu.oms9@gmail.com; 2 Department of Dental Pathology and Endodontics, Faculty of Medicine, University of Prishtina, 10000 Prishtina, Kosovo; 3 Department of Surgery, Faculty of Medicine and Dentistry, University of Salamanca, 37008 Salamanca, Spain; j.flores@usal.es; 4 Department of Dentistry, Faculty of Dental Sciences, Aldent University, 1007 Tirana, Albania; agron.meto@ual.edu.al; 5 Department of Medicine and Surgery, University of Enna “Kore”, 94100 Enna, Italy; cosimo.galletti@unikore.it (C.G.); lucafiorillo@live.it (L.F.); 6 Department of Dental Research Cell, Dr. D. Y. Patil Dental College and Hospital, Dr. D.Y. Patil Vidyapeeth, Pimpri, Pune 411018, Maharashtra, India; 7 Department of Surgery, Medicine, Dentistry and Morphological Sciences with Interest in Transplant, Oncology and Regenerative Medicine, University of Modena and Reggio Emilia, 41125 Modena, Italy

**Keywords:** antimicrobial drug delivery, biofilm, cationic lipid nanoparticles, endodontics, *Enterococcus faecalis*, nanostructured lipid carriers, solid lipid nanoparticles

## Abstract

**Background:** Persistent endodontic infections remain a significant challenge in root canal therapy, primarily due to the complexity of root canal anatomy and the formation of resistant microbial biofilms. Conventional irrigants, including sodium hypochlorite and chlorhexidine, show limited penetration into dentinal tubules and reduced efficacy against mature biofilms, contributing to treatment failure. Electrically charged lipid nanoparticles (ECLNs), such as cationic solid lipid nanoparticles, nanostructured lipid carriers, and liposomes, have emerged as potential adjunctive systems to enhance intracanal antimicrobial delivery. This focused narrative review, informed by a structured literature search, aimed to synthesize and critically evaluate preclinical and exploratory clinical evidence regarding the use of electrically charged lipid nanoparticles for antibiotic delivery and biofilm control in root canal disinfection. **Methods:** A structured literature search of PubMed, Scopus, and Web of Science (2010–2026) identified 312 records, of which 20 studies met the inclusion criteria and were included in qualitative synthesis. The majority of included studies were in vitro investigations, followed by ex vivo studies using extracted human teeth, with only a limited number of exploratory animal or clinical studies. Overall, the level of evidence was predominantly preclinical. **Results:** Across studies, ECLNs demonstrated enhanced antimicrobial efficacy compared with free antibiotics or non-charged formulations, with improved biofilm interaction, enhanced penetration into dentinal tubules, and sustained antimicrobial release. However, most investigations relied on mono-species *Enterococcus faecalis* biofilm models, and substantial heterogeneity in nanoparticle formulation and methodology was observed. Clinical evidence remains scarce. **Conclusions:** Although these findings about ECLNs suggest a promising experimental adjunct for root canal disinfection, current evidence remains largely preclinical and insufficient to support routine clinical application. Standardized formulations, clinically relevant multispecies biofilm models, and well-designed controlled clinical trials are required to establish safety, efficacy, and translational feasibility.

## 1. Introduction

Current clinical guidelines from the American Association of Endodontists (AAE) and the European Society of Endodontology (ESE) clearly state that systemic antibiotics are not indicated for routine root canal infections and are reserved only for cases with systemic involvement [1,2]. Root canal disinfection relies primarily on mechanical preparation and chemical irrigants such as sodium hypochlorite and chlorhexidine; however, biofilms and complex canal morphology still limit the efficacy of these agents [3]. Endodontic disinfection is a crucial component of root canal treatment, aiming to eliminate or reduce planktonic bacteria, thereby creating a biologically clean environment that facilitates the hermetic filling of the canal and prevents reinfection [4,5,6]. This process combines mechanical instrumentation of the canal to remove infected tissue and create access with the use of chemical irrigation agents such as sodium hypochlorite (NaOCl), chlorhexidine (CHX), and ethylenediaminotetraacetic acid (EDTA) [4,6]. Biofilms further reduce the efficacy of antibiotics, contributing to resistant bacterial phenotypes and persistent infection [7]. However, despite technological developments, complete disinfection of the root canal system remains a major clinical challenge. The anatomical complexity of the canals—including isthmuses, lateral branches, apical deltas, and deep curvatures—creates areas inaccessible to conventional irrigant solutions. Chemical interactions, such as the buffer effect of dentin, can reduce the action of agents, while the presence of the smear layer limits direct contact with microorganisms. Advanced activation modalities, such as passive ultrasonic irrigation (PUI) and laser activation (LAI), have improved penetration compared to the limited penetration of traditional irrigants [8]; however, they still do not guarantee complete elimination of the residual microbiome, showing conflicting results regarding the cleaning and disinfection rate [9,10,11,12]. This limitation is partly attributed to the EPS (extracellular polysaccharides) matrix or biofilms, which act as a barrier against antimicrobial agents [13].

Since infection of the root canal may come from caries, trauma, periodontal disease, iatrogenic reasons, or because of a failed endodontic treatment, the bacteria may establish themselves in biofilm [11]. However, in the main canal of the untreated tooth, bacteria can also be found as aggregates, coaggregates, and planktonic cells. The biofilm is defined as a sessile microbial community attached to a surface, which is composed of cells and the polymeric extracellular matrix, and depending on gene expression and growth rate, it can change its phenotype [11]. Because of the presence of the biofilm, achieving complete disinfection is one of the biggest obstacles in endodontics. These complex structures, composed of microbial communities organized and wrapped in an extracellular polymer matrix (EPS), act as a barrier to antimicrobial agents and to the host’s immune response. EPS not only limits the diffusion of active substances but also maintains a stable environment for microbial survival. Biofilm-like bacteria can be up to 1000 times more resistant than planktonic forms [13], while intracellular communication (quorum sensing) regulates the expression of virulence and resistance genes [14]. According to Xie et al. (2025), these structures not only limit drug penetration but also support the survival of persistent cells, bacterial phenotypes with minimal metabolic activity, which are extremely resistant to conventional treatments and contribute to the return of infection after treatment [15].

In this harsh environment, *Enterococcus faecalis* emerges as the most problematic pathogen, often associated with persistent infections and failures after endodontic treatment. This microorganism can survive in extreme conditions, including high pH, food deficiency, and the presence of antibiotics; penetrates deep into the tubules of dentin; and adheres to dentin collagen [12,16,17,18,19]. Although *E. faecalis* is widely used as a reference organism due to its association with persistent infections [20], root canal infections are polymicrobial in nature [21]. Recent molecular studies demonstrate that primary and secondary infections contain complex consortia of anaerobic and facultative bacteria, including *Streptococcus*, *Fusobacterium*, *Prevotella*, *Actinomyces* [22], and occasionally fungal species [23].

The remarkable resilience of *E. faecalis* under acidic stress and within host immune cells is partly due to acid tolerance genes (opp1C, copy, gnd2), which maintain intracellular pH balance and genetic stability [24]. It forms dense biofilm and modifies it to inhibit the penetration of antimicrobial agents, which makes it resistant to common intracanal disinfection procedures performed [12,16,19]. Other pathogens such as *Candida albicans*, *Fusobacterium nucleatum*, and *Actinomyces israelii* also contribute to resistance through mechanisms such as metabolic adaptation, increased flow pumps, and the production of protective substances in EPS [12]. Biofilm architecture, extracellular polymeric substance (EPS) composition, and interspecies interactions significantly increase tolerance to irrigants and antimicrobial agents compared with planktonic cells [25]. Therefore, although mono-species *E. faecalis* systems are useful for mechanistic benchmarking, the translational interpretation of nanoparticle efficacy should prioritize validation in clinically relevant mixed-species biofilm models whenever feasible [10,20,21].

Apical periodontitis (AP) remains highly prevalent worldwide and is frequently underdiagnosed because it can be asymptomatic and imaging-dependent. In a large systematic review/meta-analysis, AP was estimated to be ~52% at the individual level and ~5% at the tooth level, with AP present in ~39% of root-filled teeth, underscoring the substantial burden of post-treatment disease in endodontically treated dentitions [26,27]. Consistent with this disease burden, contemporary outcome syntheses continue to show that success rates vary substantially depending on outcome definitions (e.g., “strict” complete radiographic healing vs. “loose” criteria), follow-up duration, preoperative periapical status, and assessment modality; therefore, “failure” (often operationalized clinically as persistent/post-treatment AP or need for retreatment/surgery) should be interpreted in light of these methodological factors [28]. Recently, CBCT-based evidence further highlights that post-treatment apical periodontitis (PAP) is common in root-filled teeth and is strongly associated with anatomical/technical factors such as missed canals, reinforcing the clinical importance of improving disinfection and delivery strategies in complex biofilm-driven infections [29].

While multiple nanoparticle platforms have been explored in endodontics, this review primarily focuses on electrically charged lipid nanoparticles; non-lipid systems are discussed selectively for mechanistic comparison and contextual benchmarking. In response to persistent biological challenges and therapeutic limitations, nanotechnology has provided promising strategies to improve the efficacy of endodontic disinfection. Nanoparticles, typically defined within a size range of 1–100 nm, possess unique physicochemical properties, including high surface area-to-volume ratios, enhanced penetrability, and the capacity for surface functionalization, enabling improved interaction with microbial biofilms and dentinal structures [29,30]. These attributes allow nanoparticles to overcome anatomical and microbiological barriers within the root canal system, reaching areas inaccessible to conventional irrigant solutions and medicaments [31].

Nanoparticles can function as carriers for antibiotics, antimicrobial agents, or photosensitizers, enabling controlled and targeted delivery with enhanced antibacterial effects [32,33]. Several nanoparticle classes have been investigated in endodontics, including metallic, polymeric, chitosan-based, nanoemulsion-based, and lipid-based systems, each exhibiting distinct antimicrobial mechanisms and safety profiles [6,31,34]. Metallic nanoparticles, such as silver and zinc oxide, exert antimicrobial activity primarily through ion release and reactive oxygen species (ROS) generation, although concerns regarding cytotoxicity and long-term tissue accumulation remain [35]. Polymeric nanoparticles functionalized with antimicrobial agents have demonstrated enhanced antibiofilm activity and sustained drug release in experimental endodontic models, with biocompatibility dependent on polymer composition and degradation behavior [33,36]. Chitosan-based nanoparticles exhibit strong electrostatic interactions with bacterial membranes, contributing to biofilm disruption, though formulation variability and scalability continue to pose translational challenges [37]. Nanoemulsion-based systems have been explored to improve antimicrobial penetration and drug stability within complex root canal anatomy; however, their clinical application is limited by sensitivity to formulation balance and physical stability [31,38].

Comparative analyses indicate that nanoparticle-based approaches generally achieve greater bacterial load reduction than conventional therapies, particularly in resistant infections and mature biofilms [39]. Within this broader landscape, lipid nanoparticles—particularly cationic lipid nanoparticles (SLNs) and nanostructured lipid carriers (NLCs)—stand out due to being more biocompatible and their biodegradability, tunable surface charge, and capacity for efficient intracanal drug delivery via electrostatic interaction with negatively charged biofilms [14,40]. Unlike metallic systems that may induce oxidative stress or accumulate in tissues, lipid nanoparticles degrade into non-toxic components and can be effectively cleared, supporting their favorable safety profile [32]. Lipid-based carriers also offer versatile encapsulation of antibiotics, antiseptics, peptides, and natural bioactives, with adaptable architectures that allow precise control over drug loading, release kinetics, and surface interactions with microbial and host tissues [6,40,41].

In this review, the term electrically charged lipid nanoparticles refers specifically to cationic solid lipid nanoparticles (SLNs), nanostructured lipid carriers (NLCs), and cationic liposomes, while other nanocarriers are discussed only for mechanistic comparison. Accordingly, this review aims to: (1) describe the structural and physicochemical characteristics of electrically charged lipid nanoparticles relevant to endodontic applications; (2) analyze their mechanisms of action in biofilm disruption and antimicrobial delivery; (3) compare their performance with conventional disinfectants and non-charged formulations; and (4) identify current limitations, knowledge gaps, and future directions necessary for clinical translation.

The research question was structured using a PICO approach to define the conceptual scope of the review rather than to guide quantitative synthesis. The population included biofilm-infected root canal systems in extracted human teeth or clinical cases; the intervention involved electrically charged lipid nanoparticles used as antimicrobial carriers or adjunctive irrigants; comparators consisted of conventional endodontic disinfectants or non-charged formulations; and outcomes centered on biofilm reduction, dentinal tubule penetration, and enhanced antimicrobial delivery.

Accordingly, this review addresses the following questions: (a) In preclinical in vitro and ex vivo models, do electrically charged lipid nanoparticles improve antimicrobial penetration and biofilm reduction compared with free antimicrobial agents or non-charged formulations? (b) What is the current level of clinical evidence supporting their use in root canal disinfection?

## 2. Materials and Methods

### 2.1. Study Design

This manuscript is a focused narrative review informed by a structured, reproducible literature search, designed to critically synthesize preclinical and exploratory clinical evidence on electrically charged lipid nanoparticles for root canal disinfection. The review followed established methodological principles for narrative syntheses informed by systematic search strategies while acknowledging that heterogeneity of study designs precluded quantitative meta-analysis. Accordingly, the review should be interpreted as a narrative synthesis informed by systematic search and screening procedures, rather than as a formal systematic review or meta-analysis.

### 2.2. Literature Search Strategy

A comprehensive literature search was conducted in PubMed, Scopus, and Web of Science to identify relevant studies published between January 2010 and January 2026. This timeframe was selected to capture the emergence and development of lipid nanoparticle platforms in biomedical and dental research.

The search strategy combined Medical Subject Headings (MeSH) and free-text keywords related to lipid nanoparticles, electrical charge, antimicrobial delivery, and endodontic biofilms. The primary search strings included:

- (“lipid nanoparticles” OR “solid lipid nanoparticles” OR “nanostructured lipid carriers” OR “cationic liposomes”) AND (“root canal” OR “endodontic” OR “endodontic disinfection” OR “biofilm”);
- (“cationic nanoparticles” OR “electrically charged nanoparticles” OR “zeta potential” OR “surface charge”) AND (“*Enterococcus faecalis*” OR “multispecies biofilm”);
- (“nanoparticle drug delivery” AND “endodontics”).

Search strings were adapted for each database. Reference lists of relevant review articles were additionally screened to identify eligible studies not captured in the electronic search.

### 2.3. Study Selection

All retrieved records were imported into a reference management software, and duplicates were removed. Two authors independently screened titles and abstracts to assess relevance. Full-text articles were subsequently evaluated against predefined inclusion and exclusion criteria. Disagreements were resolved through discussion and consensus.

### 2.4. Inclusion Criteria

Studies were included if they met all of the following criteria:

1. Investigated electrically charged lipid-based nanocarriers, including solid lipid nanoparticles (SLNs), nanostructured lipid carriers (NLCs), cationic liposomes, or closely related lipid-based systems;
2. Evaluated antimicrobial delivery, biofilm reduction, or dentinal tubule penetration in the context of endodontic disinfection;
3. Employed in vitro, ex vivo, animal, or clinical models relevant to root canal infections;
4. Reported at least one quantitative or semi-quantitative outcome, such as bacterial viability, biofilm biomass, penetration depth, release kinetics, or zeta potential;
5. Published in peer-reviewed journals and written in English.

### 2.5. Exclusion Criteria

Studies were excluded if they met any of the following criteria (Table S1):

1. Focused exclusively on non-lipid nanoparticles (e.g., metallic, polymeric, chitosan-based, or nanoemulsion systems), unless included for direct mechanistic comparison;
2. Unrelated to endodontic disinfection or antimicrobial activity;
3. Lacked characterization of surface charge or electrical properties;
4. Were conference abstracts, editorials, or non-peer-reviewed publications;
5. Represented duplicate datasets.

### 2.6. Data Extraction

Data were independently extracted by two reviewers using a standardized form. Extracted variables included:

- Type of lipid nanocarrier (SLN, NLC, liposome);
- Particle size, polydispersity index, and zeta potential;
- Lipid composition and surface-modifying agents;
- Encapsulated antimicrobial or active compound;
- Experimental model (in vitro, ex vivo, animal, or clinical);
- Biofilm model (mono-species or multispecies);
- Antimicrobial outcomes (CFU reduction, biofilm biomass reduction);
- Penetration depth into dentinal tubules;
- Drug release characteristics and stability parameters.

### 2.7. Quality Assessment

Given the predominance of preclinical studies and the absence of standardized risk-of-bias tools for nanomaterial-based in vitro research, a qualitative methodological appraisal was performed.

A structured checklist adapted from CONSORT (for reporting transparency), ARRIVE (for experimental rigor in biological models), and PRISMA principles (for study selection transparency) was applied. These frameworks were used to guide assessment of reporting completeness and methodological transparency rather than to indicate formal compliance. Studies were evaluated across the following domains:

1. Completeness of nanoparticle characterization (size distribution, zeta potential, composition, stability);
2. Validity and clinical relevance of biofilm models;
3. Clarity and reproducibility of experimental methods;
4. Quantitative outcome reporting and statistical analysis;
5. Translational relevance to endodontic practice.

Studies with comprehensive nanoparticle characterization and standardized experimental protocols were considered at lower risk of bias, whereas studies lacking essential reporting details were considered at higher risk. No numerical scoring system was applied; instead, studies were qualitatively categorized as having lower or higher methodological transparency based on reporting completeness. Identified limitations were incorporated into the narrative synthesis.

### 2.8. Data Synthesis

Due to heterogeneity in nanoparticle formulations, physicochemical characteristics, antimicrobial payloads, biofilm maturation protocols, and outcome reporting, quantitative meta-analysis was not feasible. Therefore, a structured qualitative narrative synthesis was conducted. Findings were organized thematically according to nanoparticle type (solid lipid nanoparticles, nanostructured lipid carriers, cationic liposomes), physicochemical properties (particle size, zeta potential), antimicrobial efficacy, experimental model (in vitro, ex vivo, animal, or exploratory clinical), biofilm model (mono-species vs. multispecies), and primary outcome measures (CFU reduction, biofilm biomass reduction, dentinal tubule penetration depth, release kinetics, cytocompatibility).

Findings were synthesized comparatively across these domains to identify consistent patterns, methodological limitations, and translational gaps.

**Figure 1:**
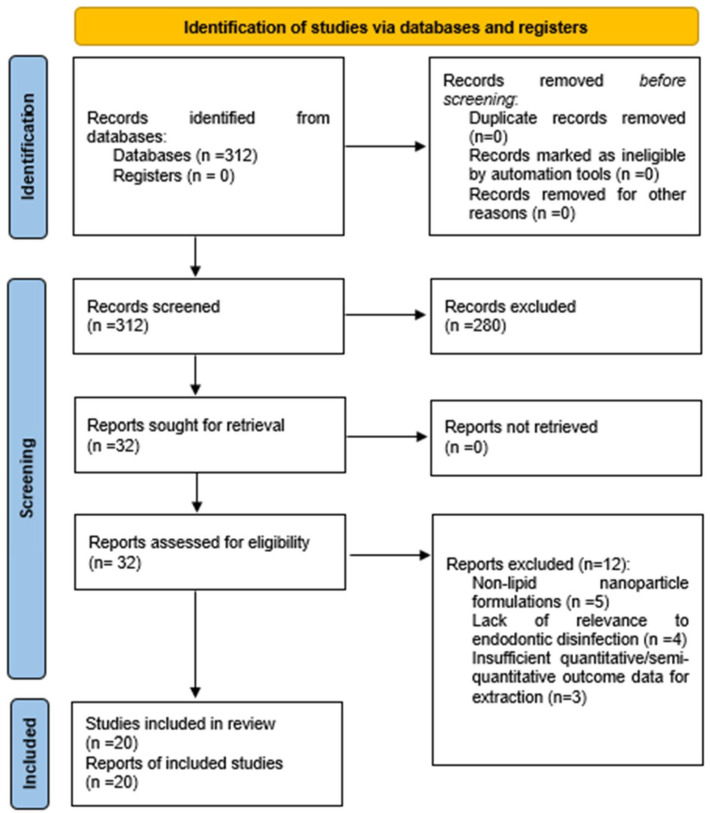
PRISMA 2020 Flow Diagram of the Study Selection Process.

The study selection process was conducted according to the PRISMA 2020 guidelines [42], and the detailed screening procedure is presented in the flow diagram (Figure 1).

## 3. Results

### 3.1. Study Selection

The database search identified 312 records across PubMed, Scopus, and Web of Science. After the removal of 114 duplicates, 198 unique records were screened by title and abstract. Of these, 41 articles were assessed for eligibility, and 15 were excluded for predefined reasons, including non-lipid nanoparticle formulations (n = 5), lack of relevance to endodontic disinfection (n = 4), absence of surface charge characterization (n = 2), and conference abstracts lacking sufficient data (n = 2). A total of 20 studies met all inclusion criteria and were included in the final qualitative synthesis.

### 3.2. Characteristics of Included Studies

The 20 included studies comprised original experimental investigations of electrically charged lipid nanoparticles in endodontic models; review articles and non-lipid nanocarrier studies were cited for contextual or mechanistic discussion only. The majority of included studies were in vitro investigations, followed by ex vivo studies using extracted human teeth or dentin blocks, with only a single exploratory animal study, with no human clinical trials identified.

Most studies evaluated electrically charged lipid nanoparticles, including solid lipid nanoparticles (SLNs), nanostructured lipid carriers (NLCs), and cationic liposomes. Particle sizes typically ranged from 80 to 250 nm, with reported positive zeta potentials between +20 and +45 mV, values considered sufficient to promote electrostatic interaction with bacterial membranes and biofilm matrices.

Comparative synthesis across nanoparticle types revealed that studies with clearly defined surface charge and stability parameters more consistently reported enhanced antimicrobial outcomes. However, heterogeneity in model selection and outcome definitions limited direct inter-study comparability. Of the 20 included reports, 15 were primary experimental investigations, and 5 were narrative or background review articles. The level of clinical evidence was assessed for primary experimental studies only (Table 1). The individual studies contributing to each category are detailed in the corresponding summary tables.

### 3.3. Electrically Charged Lipid Nanoparticles: Structure, Function, Principles, and Design

Electrically charged lipid nanoparticles (Solid Lipid Nanoparticles—SLN and Nanostructured Lipid Carriers—NLC) represent an advanced platform for the delivery of antimicrobial agents in endodontic therapy, thanks to the combination of more biocompatible nature, the capacity to encapsulate both hydrophobic and hydrophilic molecules, and the ability to provide controlled drug release [1,14]. Ahsan et al. (2024) emphasize that these systems are capable of carrying a wide number of therapeutic compounds, including antibiotics, antimicrobial adjuvants, biofilm-degrading enzymes, and agents with photodynamic action [14]. This variety gives lipid nanoparticles an active role in endodontic therapy, acting not only as passive carriers but also as agents that interact directly with the microbial environment. Lipid nanoparticles, typically composed of biodegradable and biocompatible lipids, such as triglycerides, phospholipids, and non-ionic surfactants, feature a solid or semi-solid lipophilic core surrounded by a stabilizing outer layer. As Tenchov et al. (2021) and Hou et al. (2021) describe, this configuration protects encapsulated agents from premature degradation and enables controlled release; in endodontic therapy, these properties can be leveraged to achieve a sustained delivery of antimicrobials within the infected root canal system [30,43].

SLNs are lipid-based nanocarriers composed of a solid lipid matrix that can exhibit neutral, anionic, or cationic surface charge depending on lipid composition and surface-modifying excipients. In endodontics, cationic lipid nanoparticles (LNPs) have gathered considerable attention due to their strong electrostatic attraction to negatively charged surfaces, including bacterial membranes, extracellular matrix components, and dentinal walls [32,40]. This further enhances their ability to localize at the site of infection, adhere to biofilms, and penetrate deep into bacterial communities [44]. This charge is typically introduced by incorporating cationic lipids, which increase interaction with negatively charged bacterial membranes and with the extracellular matrix of biofilms [15,44]. Cationic variants of electrically charged lipid nanoparticles have become a focal point in nanomedical applications due to their enhanced interaction with negatively charged bacterial membranes and biofilm components [1,14]. This subclass includes solid lipid nanoparticles (SLNs), nanostructured lipid carriers (NLCs), and cationic liposomes, all of which offer favorable characteristics such as biocompatibility, encapsulation of both hydrophilic and hydrophobic drugs, and sustained release [6,32]. The main structural categories of this subclass are represented in the Table 2.

The positive surface charge (zeta potential) facilitates electrostatic attraction to microbial surfaces, promoting adhesion and intracellular delivery of active agents [45,46,47]. For instance, SLNs with zeta potentials ranging from +25 to +45 mV demonstrate improved retention within infected dentin and superior biofilm disruption [48]. Table 3 below summarizes various electrically charged lipid nanoparticle systems and their observed antibiofilm effects.

The surface charge, quantified by zeta potential, is one of the most critical determinants of how a nanoparticle is going to perform. Cationic LNPs generally exhibit zeta potentials ranging from +20 to +50 mV, sufficient to promote bacterial membrane binding, disrupt electrostatic equilibrium in biofilms, and facilitate uptake into negatively charged dentin structures [6,32]. The presence of such a positive electric charge in cationic lipid nanoparticles, besides enabling strong electrostatic interactions with negatively charged surfaces of microbial membranes and biofilm matrix, facilitates their penetration into the biofilm structure and increases the direct contact with pathogens [14,46,54]. Importantly, the mechanism of this technology makes it a promising approach for overcoming the challenges posed by resistant biofilms, especially those of *E. faecalis* [14,19,40,46]. Moreover, unlike neutral lipid nanoparticles, which have limited interaction with microbial surfaces, cationic formulations harness the electrical charge to maximize affinity to pathogens and increase disinfection efficiency [16,44,55].

### 3.4. Antimicrobial Efficacy Against Endodontic Biofilms

Across studies, electrically charged lipid nanoparticles demonstrated enhanced antimicrobial efficacy compared with free antimicrobial agents or non-charged formulations. This effect was consistently observed in mono-species biofilm models, particularly those involving *E. faecalis*.

Reported outcomes included:

- Significant reductions in colony-forming units (CFU);
- Decreased biofilm biomass;
- Prolonged antimicrobial activity compared with conventional irrigants or free drug solutions.

Several studies reported that enhanced antimicrobial performance was associated with electrostatic adhesion to negatively charged bacterial surfaces, which increased nanoparticle retention within the biofilm and facilitated closer contact between the antimicrobial payload and microbial cells.

#### Mechanism of Action of Lipid Nanoparticles

Insights from studies on inorganic nanoparticles, such as zinc oxide (Sirelkhatim et al., 2015), emphasize the importance of physicochemical parameters, including particle size, surface charge, and ROS generation, in dictating antibacterial efficacy [56]. These principles similarly inform the design of electrically charged lipid nanoparticles for endodontic therapy.

Cationic SLNs are typically formulated by incorporating surfactants such as DOTAP or quaternary ammonium compounds, which impart a positive surface charge that enhances adhesion to bacterial membranes and to the anionic components of the EPS matrix [40,57]. The mechanism of action of these nanoparticles is based on electrostatic targeting of negatively charged bacterial membranes, fusion of nanoparticles with cell membranes to increase antibiotic uptake, and controlled release that maintains therapeutic levels for extended periods of time. These mechanisms not only increase the antimicrobial effect but also allow for a reduction in total drug dose, thereby reducing toxicity and impact on the healthy microbiota [6,14]. Permeability into biofilm structures is the third critical element of this therapeutic triad. Biofilms act as a strong physical and biochemical barrier to traditional drugs, but cationic SLNs overcome these barriers through electrostatic interactions with the EPS matrix and bacterial membranes, enhancing disruption of the biofilm and stable pathogen elimination [39].

**Figure 2:**
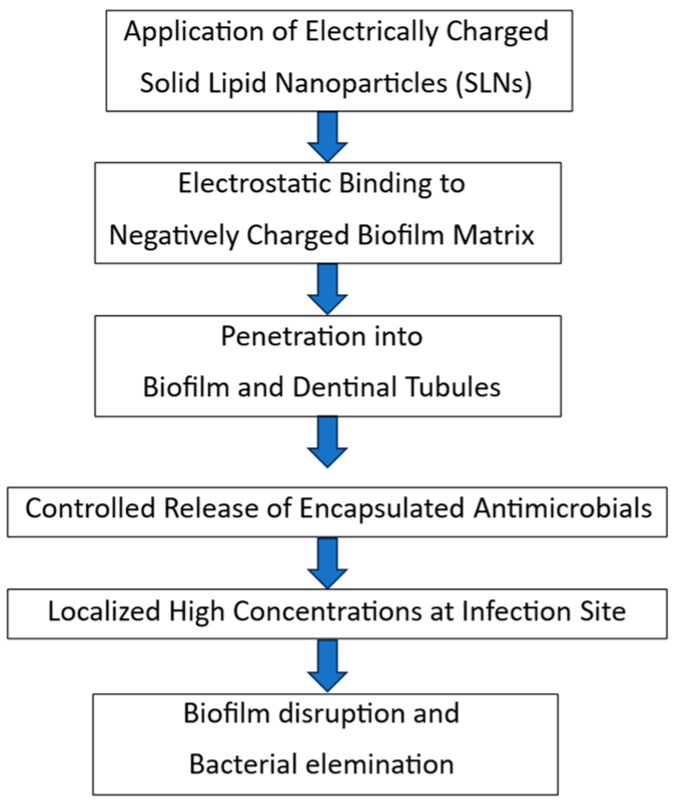
Mechanism of action of electrically charged solid lipid nanoparticles (SLNs) in endodontic therapy. Positively charged SLNs bind electrostatically to the negatively charged biofilm matrix, penetrate biofilm structures and dentinal tubules, and provide controlled release of antimicrobials. This results in high local concentrations at the infection site, enhancing biofilm disruption and bacterial elimination.

Figure 2 illustrates the proposed mechanism of action of electrically charged SLNs, highlighting their electrostatic interaction with the biofilm matrix, penetration into dentinal tubules, controlled antimicrobial release, and the resulting high concentration of the drug at the infection site. These mechanistic advantages underscore their strong therapeutic potential in endodontic disinfection, while also emphasizing the complexity and rigorous validation still required before clinical translation and widespread adoption.

Studies have shown that different formulations of electrically charged lipid nanoparticles have demonstrated significant antibiofilm effects and improved drug delivery. The results suggest that cationic lipid nanoparticle technology can be integrated as a supportive or alternative strategy to traditional disinfection methods, offering potential for reducing the recurrence of infections and improving clinical outcomes [32,48].

### 3.5. Penetration into Dentinal Tubules

Several studies specifically evaluated dentinal tubule penetration using dentin block or extracted tooth models. Electrically charged lipid nanoparticles consistently demonstrated greater penetration depth than free antimicrobial agents, reaching depths of several hundred micrometers into dentinal tubules. Cationic formulations showed superior retention within dentin compared with neutral or anionic counterparts, likely due to electrostatic attraction to the negatively charged dentinal matrix. These findings were consistently observed in studies evaluating surface charge-dependent formulations.

With mean diameters under 200 nm, SLNs have demonstrated the capacity to penetrate dentinal tubules (1–3 µm wide) in experimental models, extending antimicrobial activity into areas inaccessible to conventional endodontic measures [31]. These mechanisms lead to improved biofilm eradication [44], sustained and controlled drug release properties [57], and deep penetration into biofilm structures [44] and into dentinal tubules when delivered through specialized SLN formulations [40,58], and, when combined with activation techniques such as PUI, LAI, and aPDT, they may cause a synergistic effect [32]. Evidence from photodynamic therapy research shows that lipid-based vesicles incorporating cationic components (e.g., invasomes /liposomes with DOTAP + ethanol) can significantly reduce *E. faecalis* within dentinal tubules, achieving up to 5.7 log reduction in vitro [59]. Moreover, photoactivated SLN formulations, such as curcumin-loaded particles combined with photodynamic therapy, have demonstrated enhanced antimicrobial effect against *E. faecalis* biofilms in dentinal tubule models [40].

### 3.6. Drug Delivery and Release Characteristics

Lipid nanoparticle systems enabled controlled and sustained release of encapsulated antimicrobial agents, including antibiotics, antiseptics, and photosensitizers. Compared with free drugs, nanoparticle-based formulations reduced burst release and maintained therapeutic concentrations over extended periods.

Several studies reported that sustained release contributed to reduced bacterial regrowth and improved antibiofilm efficacy, supporting the use of electrically charged lipid nanoparticles as intracanal drug delivery systems rather than simple antimicrobial substitutes.

Moreover, the type and concentration of cationic surfactants, such as CTAB, DDAB, or quaternary ammonium compounds, affect both antimicrobial activity and biocompatibility [40]. Importantly, LNPs can be engineered for stimuli-responsive drug release, responding to environmental cues such as pH changes or enzymatic activity. In the acidic conditions found in infected root canals, certain LNPs disintegrate and release their antimicrobial payload in a controlled manner, optimizing therapeutic efficacy while minimizing systemic toxicity [31,41]. Solid lipid nanoparticles are distinguished from other lipidic nanocarriers by their solid lipid matrix, which protects encapsulated drugs from premature degradation, while providing controlled drug release, because of their ability to remain stable at body temperature [30,41].

Drug release from SLNs is governed by lipid crystallinity and the distribution of active agents within the lipid matrix, preventing burst release and enabling gradual antimicrobial delivery [30,40]. This gradual release helps maintain stable therapeutic concentrations, prevents bacterial recolonization, and reduces the need for repeated applications or high doses, thereby improving therapeutic safety [13]. Ferreira et al. (2025) demonstrated that curcumin-loaded SLNs, when incorporated into hydrogels, significantly improve drug solubility and provide sustained release over 72 h, with enhanced antibiofilm effect against *E. faecalis* under photoactivation, while maintaining cytocompatibility [60]. Although not strictly SLNs, these findings reinforce the principle that electrically charged lipid carriers can enhance penetration into the root canal system and provide superior antimicrobial outcomes compared to conventional irrigant solutions.

While controlled release profiles of ECLNs have been characterized primarily under static in vitro conditions, release behavior within the dynamic intracanal environment may differ substantially. In clinical root canal systems, irrigant exchange, fluid shear stress, and activation techniques such as passive ultrasonic activation (PUI) or laser activation (LAI) generate complex hydrodynamic patterns that can influence nanoparticle dispersion and drug diffusion [3,9]. Increased fluid turnover may theoretically accelerate drug clearance from the canal space, potentially reducing sustained antimicrobial exposure compared with closed in vitro release models. Conversely, electrostatic adhesion of cationic lipid nanoparticles to negatively charged dentinal walls and biofilm matrices may partially mitigate rapid washout, supporting localized retention and prolonged antimicrobial contact [6,14].

The phenomenon of burst release, frequently described in lipid-based nanocarriers due to surface-associated drug fractions and matrix characteristics [30,40], may provide an initial high antimicrobial concentration that enhances early biofilm disruption. However, excessive burst release could potentially shorten the duration of effective drug levels and may increase local cytotoxic risk if not carefully optimized. Although several included studies reported sustained release over 24–72 h under laboratory conditions [40,60], data evaluating release kinetics under simulated intracanal fluid dynamics or clinically relevant irrigation scenarios remain limited. Therefore, extrapolation of controlled-release behavior to in vivo root canal conditions should be interpreted cautiously.

Arabestani et al. (2024) highlight the flexibility of these systems in adapting to different therapeutic molecules, as well as their potential for personalized treatment depending on the type and severity of the infection [57]. Thanks to their natural composition and compatibility with human tissues, lipid nanoparticles are considered biocompatible and promising for clinical translation into the endodontic environment [57]. In addition, Mondal et al. (2024) and Raura et al. (2020) emphasize the advantages of lipid nanoparticles over traditional methods, including better control of active substance release, reduced systemic exposure, and a lower systemic exposure and improved pharmacokinetic control, as described in broader antimicrobial nanoparticle research [34,61], with potential relevance for intracanal applications.

### 3.7. Biofilm Models and Microbial Diversity

Most studies employed mono-species biofilm models, predominantly using *E. faecalis*. Only a limited number investigated mixed-species biofilms despite the polymicrobial nature of clinical endodontic infections.

Studies utilizing more complex biofilm models generally reported reduced, but still significant, antimicrobial efficacy compared with mono-species systems, highlighting the importance of model selection when interpreting preclinical results. Across the included studies, the majority employed mono-species biofilm models, predominantly based on *Enterococcus faecalis*, while only a limited subset investigated mixed-species biofilms. The primary concentration in *E. faecalis* and the lack of testing for biofilms, including fungi such as *C. albicans*, limit clinical relevance [62,63,64].

### 3.8. Comparison with Conventional Disinfection Strategies

When compared with standard endodontic disinfectants such as NaOCl and CHX, electrically charged lipid nanoparticles demonstrated:

- Improved biofilm penetration;
- Enhanced retention within dentinal tubules;
- Lower cytotoxicity in reported biocompatibility assays;
- Prolonged antimicrobial action.

Across studies, electrically charged lipid nanoparticles were evaluated primarily as adjunctive systems rather than standalone replacements for conventional irrigants. However, when comparing ECLNs with conventional irrigation solutions such as NaOCl and CHX, experimental heterogeneity must be carefully considered. Across studies, substantial variability exists in irrigant concentration, contact time, biofilm maturation stage, activation protocols (e.g., passive ultrasonic activation or laser activation), and experimental models (e.g., dentin block vs. whole tooth systems) [3,9,10,25]. These methodological differences can significantly influence antimicrobial outcomes and penetration depth. For instance, mature biofilms demonstrate greater tolerance than early-stage biofilms, and variations in exposure time or irrigant concentration may either amplify or underestimate comparative efficacy. Therefore, direct comparisons between nanoparticle-based systems and conventional irrigants should be interpreted within the specific experimental context of each study.

Comparing efficacy between conventional and nanoparticle-based treatments represents an important dimension in evaluating new advances in endodontic therapy. To highlight the differences between traditional and emerging therapeutic options, Table 4 summarizes key characteristics such as biofilm penetration, toxicity, and clinical applicability across various endodontic treatment strategies. This comparative overview emphasizes the advantages of lipid-based and nanoparticle-supported formulations in overcoming limitations associated with conventional approaches.

While traditional treatments have been the standard of care for decades, recent developments in nanotechnology are showing high potential to improve clinical outcomes, especially in combating stable bacterial biofilms.

Conventional therapies, based on systemic antibiotics and intracanal disinfectants such as NaOCl and CHX, show limited efficacy against microorganisms embedded in biofilms. As Salas-Orozco et al. (2019) note, these treatments encounter problems such as bacterial resistance, local toxicity, and insufficient penetration into inaccessible areas of the root canal system [75]. Orozco et al. (2025) further point out that despite their rapid initial action, traditional disinfectants have difficulty eradicating mature biofilms and may permit microbial recolonization [65]. In contrast, nanoparticle-based approaches have gained attention for their advanced targeted antimicrobial delivery [32,76]. In a comparative study, it was reported that lipid and metal nanoparticles were significantly more effective than traditional treatments in eliminating *E. faecalis* and other pathogens of the endodontic system. Their superior efficacy is attributed to enhanced penetration, stabilization of active ingredients, and the potential for controlled drug release within the infected site [32].

From a practical perspective, Diogo et al. (2023) review the applications of liposomes, niosomes, and transferosomes, three common forms of lipid nanoparticles, in root canal disinfection [77]. They highlight that these carriers show promising effectiveness against *E. faecalis*, a bacterium known for its resistance to colonize inaccessible canal areas [77].

Table 5 presents an overview of the different cationic nanoparticle formulations applied in endodontic therapy for biofilm control. Reported types include Solid Lipid Nanoparticles (SLNs), Nanostructured Lipid Carriers (NLCs), liposomes, nanoemulsions, and functionalized metal nanoparticles, which exhibit positive zeta potential (+15 to +45 mV), a typical size of 20–250 nm, and ability to encapsulate or release antimicrobial agents (antibiotics, antiseptics, photosensitizers) or exploit their antimicrobial properties, which stem from the composition and structure of the material without the need for the addition of other active agents. Results from in vitro, ex vivo, and in vivo studies show a strong reduction in *E. faecalis* biofilms and other pathogens, improved penetration into dentin structures, sustained drug release, and synergistic effects when combined with photodynamic therapy or other activation methods [48,78]. To improve comparative interpretation, nanoparticle physicochemical properties, experimental models, and primary antimicrobial outcomes were consolidated into unified comparative tables.

Finally, because SLNs degrade into physiological lipids, they are generally associated with lower cytotoxic risks than metallic NPs, making them safer for periapical tissues compared to NaOCl or high-dose CHX [6,32]. Lipid nanoparticles are generally considered more biocompatible than metallic nanoparticles due to their biodegradability; however, comparative safety data in endodontic tissues remain limited [13].

### 3.9. Methodological Heterogeneity and Risk of Bias

Substantial heterogeneity was observed across included studies with respect to:

- Nanoparticle composition and surface modifiers;
- Particle size and zeta potential;
- Biofilm maturation times;
- Outcome measures and reporting standards.

Many studies lacked full nanoparticle characterization or standardized biofilm protocols, contributing to a moderate to high risk of bias and limiting direct comparison between studies. Based on the qualitative appraisal, studies demonstrating comprehensive nanoparticle characterization (including size distribution, zeta potential, and stability data) and standardized biofilm maturation protocols were considered to exhibit higher methodological transparency. In contrast, studies lacking full physicochemical reporting or employing non-standardized biofilm models were considered to present a higher potential risk of bias. Overall, reporting completeness varied substantially across included studies.

#### Identifying Gaps and the Need for a Systematic Review

Although electrically charged lipid nanoparticles have shown high potential in medicine, their application in endodontics remains limited and largely based on in vitro or ex vivo studies, with little standardized clinical evidence [32,33]. Studies often differ in particle size, zeta potential, active agent concentration, and biofilm measurement methodologies, making it difficult to compare between them. To date, there is a lack of a systematic review comprehensively assessing the effectiveness of electrically charged nanoparticles in antibiotic delivery and biofilm control; existing reviews highlight knowledge gaps but stop short of systematic evaluation [15,77]. For future research, systematic reviews identifying gaps and providing clear guidance following PRISMA guidelines should be conducted.

Table 6 provides a summary of recent studies (2022–2025) exploring nanoparticle systems relevant to endodontics, with a focus on lipid-based and electrically charged carriers. The table highlights their experimental models, particle characteristics, and key findings in relation to antimicrobial efficacy and biofilm disruption.

## 4. Discussion

Overall, most studies demonstrated a moderate to high risk of bias due to a lack of standardized biofilm models, the absence of blinding, and incomplete nanoparticle characterization, limiting the strength of translational conclusions. This narrative review highlights promising preclinical trends but does not provide conclusive evidence of clinical efficacy due to substantial heterogeneity in study designs, formulations, and outcome measures. The physicochemical features of SLNs enable targeted drug distribution, sustained release, and improved penetration into biofilm and dentinal tubules, directly addressing the limitations of systemic antibiotics and traditional irrigants [41,47,57,87]. Compared with standard therapies, cationic SLNs enhance the elimination of *E. faecalis* and reduce recolonization risks by overcoming biofilm-mediated protection [32,39,40]. These advantages are reflected in experimental findings showing that nanocarriers can enhance antimicrobial delivery compared with free drugs in controlled laboratory models. Although not developed for endodontic applications, charge-switchable lipid nanoparticles described by Liu et al. (2023) illustrate how environmental responsiveness and surface modulation could inspire future targeted intracanal delivery systems [89].

Numerous in vitro and preclinical studies have demonstrated the antimicrobial efficacy of lipid nanoparticles, especially positively charged systems that interact electrostatically with bacterial membranes and the extracellular polymeric matrix [44,87]. Fungi are not considered core constituents of most endodontic infections; however, systematic evidence indicates that fungal species, most commonly *Candida* spp., can be detected in a subset of cases, particularly in persistent or secondary infections and in mixed microbial communities [23]. Because the present review focuses on ECLNs platforms and the dominant evidence base is bacterial (often *E. faecalis*), fungal findings are interpreted cautiously. Nonetheless, the documented ability of *Candida* spp. to form biofilms and interact within polymicrobial communities supports the need for future experimental models incorporating bacterial–fungal interactions when evaluating advanced antimicrobial delivery systems for refractory biofilm-associated disease [23,63,64]. Despite these promising laboratory findings, clinical translation remains limited, with most formulations still confined to experimental or developmental stages and evidence largely restricted to controlled models rather than patient trials [6]. Beyond penetration and controlled release, advanced delivery approaches are being explored to further optimize antimicrobial efficacy. Miranda et al. (2023) and Panthi et al. (2024) emphasize the potential of targeted delivery mechanisms, such as ligand-functionalized and stimulus-responsive carriers, which improve drug selectivity and reduce side effects [6,87]. These findings align with the analysis of the current review, indicating that such nanocarriers can enhance pharmacokinetic control and help reduce microbial recolonization after treatment [6,87]. While some nanocarriers, such as silver nanoparticles and chitosan-based ones, have gained broader approval for antimicrobial use, specialized electrically charged SLNs tailored for root canal therapy lack standardization, regulatory approval, and clinical integration [40,41]. Recent research highlights that the successful application of electrically charged SLNs in clinical settings will rely on their integration into established endodontic practices. While incorporating them into irrigants, intracanal medicaments, and root canal sealers has yielded encouraging in vitro results, challenges persist regarding reproducibility and formulation stability [39,49]. Furthermore, the compatibility of these SLNs with existing clinical procedures, including mechanical instrumentation and widely utilized irrigants like NaOCl and CHX, requires thorough assessment to guarantee safety and effectiveness before their routine use.

This gap between experimental potential and clinical adoption highlights the need for translational research, including large-scale multicenter trials, rigorous safety assessments, and clear regulatory pathways [39,77]. Beyond biological validation, translational barriers include regulatory approval pathways, large-scale manufacturing reproducibility, cost-effectiveness compared with established irrigants, and compliance with Good Manufacturing Practice (GMP) standards. The classification of nanoparticle-based intracanal medicaments as pharmaceutical, device, or combination products may further complicate approval processes. Recent reviews further stress the importance of customized delivery systems sensitive to the microbiological and anatomical variability of root canals, as well as closer collaboration between pharmaceutical scientists and oral health professionals to adapt nanotechnologies to existing clinical standards [6,15,90].

The literature strongly supports the unique advantages of cationic SLNs. Their positive surface charge enhances adhesion to negatively charged bacterial membranes and dentinal substrates, while their lipid matrix protects the encapsulated drug and provides controlled release. These properties translate into improved biofilm disruption, reduced systemic dosing, and lower toxicity compared to conventional irrigant solutions [32,39,40]. Comparative evidence shows that, although other nanocarrier systems (e.g., liposomes, polymeric nanoparticles, metallic particles) share some mechanistic benefits, SLNs are distinguished by their biocompatibility, degradability into physiological lipids, and stability at body temperature [30,41].

While lipid-based nanoparticles are widely regarded as more biocompatible than metallic nanocarriers, important safety considerations remain for endodontic applications. In cases of inadvertent periapical extrusion, conventional irrigants such as sodium hypochlorite are associated with well-documented cytotoxic and inflammatory complications [66]. Although ECLNs may present a more favorable cytocompatibility profile based on preclinical studies [13,40], direct evidence assessing periapical tissue response following extrusion is currently lacking. In addition, long-term degradation behavior and the possibility of local tissue accumulation under clinically relevant conditions have not been systematically investigated. Most available safety data derive from controlled in vitro assays or short-term experimental models [13,40], which do not fully replicate host immune responses and periapical healing dynamics. Therefore, comprehensive in vivo and long-term safety studies are required before definitive conclusions regarding clinical safety can be drawn.

Nonetheless, the clinical translation of these advantages is far from straightforward, as several biological and practical barriers remain. Although several experimental studies, particularly those evaluating cationic liposomal and selected SLN-based systems, have demonstrated enhanced penetration into dentinal tubules [40,50,53], these findings must be interpreted in light of clinical modifiers that influence dentin permeability. The presence of the smear layer, generated during mechanical instrumentation, may act as a physical barrier limiting nanoparticle diffusion unless adequately removed with chelating agents such as EDTA [4]. Furthermore, anatomical variability and structural changes in dentin may reduce tubule permeability under clinical conditions compared with standardized laboratory dentin block models [11,25].

In addition, canal irrigation dynamics play a critical role in intracanal distribution. Fluid flow patterns, irrigant exchange, and activation techniques such as passive ultrasonic activation (PUI) and laser activation (LAI) significantly influence the depth and uniformity of irrigant penetration [3,9,10]. Most nanoparticle penetration studies have been conducted under controlled in vitro or ex vivo conditions that do not fully replicate the complex hydrodynamic environment of the clinical root canal system.

Importantly, in vivo data on nanoparticle penetration into dentinal tubules remain scarce. The majority of available evidence is derived from simplified experimental models with standardized canal anatomy, absence of host immune factors, and controlled irrigation protocols. Therefore, while penetration results are promising, their direct clinical extrapolation should be approached cautiously.

Recent analyses indicate that even advanced nanocarrier systems may demonstrate limited penetration in dense or mature biofilms, potentially fostering resistant phenotypes [16,31,91]. In addition, variability in dentinal tubule anatomy, host immune response, and biofilm composition further complicates translation into predictable clinical outcomes.

In conclusion, electrically charged SLNs may represent a promising frontier in endodontic disinfection by combining controlled drug delivery, biocompatibility, and enhanced biofilm penetration. However, translation from bench to bedside remains constrained by the predominance of simplified preclinical models, as clinical endodontic infections are polymicrobial and structurally more complex. This represents a major translational gap, underscoring the need for future experimental standards that incorporate multispecies biofilm consortia, extended maturation periods, and dentin block or whole-tooth models to better establish standardized formulations, long-term safety data, and integration into clinical protocols. Future directions may also include smart, patient-specific systems exploiting targeting ligands or stimuli-responsive release, supported by multidisciplinary collaboration among dentists, nanoscientists, pharmacologists, and engineers. Importantly, electrically charged SLNs should be viewed as adjunctive systems that enhance, rather than replace, mechanical instrumentation and conventional irrigation protocols.

### Limitations and Future Research Directions

Despite the promising findings synthesized in this review, several important limitations must be acknowledged. First, the available evidence is predominantly preclinical. The majority of included studies were in vitro investigations, often employing simplified mono-species biofilm models, primarily based on *Enterococcus faecalis*. While these models are useful for mechanistic evaluation, they do not fully replicate the polymicrobial complexity, anatomical variability, and host-related factors present in clinical root canal infections. Only a limited number of exploratory animal studies were identified, and no randomized controlled trials evaluating ECLNs for root canal disinfection were found.

Second, substantial methodological heterogeneity was observed across studies. Variability in nanoparticle composition, surface charge, particle size, encapsulated agents, biofilm maturation protocols, and outcome measures limits direct inter-study comparability and precludes quantitative synthesis. Inconsistent reporting of physicochemical parameters, including stability data and release kinetics, further complicates reproducibility and translational interpretation.

Third, most release kinetics and penetration data were generated under static laboratory conditions, which may not accurately reflect the dynamic intracanal environment influenced by irrigation protocols, fluid shear stress, and activation techniques such as passive ultrasonic activation or laser activation.

Fourth, the merging nature of nanotechnology-based endodontic therapies introduces potential publication bias, as studies reporting positive antimicrobial outcomes may be more likely to be published than those demonstrating neutral or negative findings.

Future research should prioritize:

(1) Standardized nanoparticle characterization and reporting frameworks;
(2) Clinically relevant multi-species biofilm models with extended maturation periods;
(3) Evaluation under simulated intracanal fluid dynamics;
(4) Long-term in vivo safety assessment, including periapical tissue response;
(5) Well-designed randomized clinical trials to establish safety, efficacy, optimal dosing protocols, and integration into conventional irrigation strategies.

Addressing these limitations is essential to bridge the current translational gap between promising laboratory data and routine clinical implementation.

## 5. Conclusions

ECLNs, including solid lipid nanoparticles, nanostructured lipid carriers, and cationic liposomes, show considerable promise as adjunctive systems for root canal disinfection. Preclinical evidence indicates that their positive surface charge and lipid-based structure enhance antimicrobial delivery, biofilm disruption, and penetration into dentinal tubules compared with conventional irrigants and free drugs. Despite these encouraging findings, the available evidence is predominantly preclinical and highly heterogeneous. Most studies rely on mono-species biofilm models and lack standardized nanoparticle characterization, limiting clinical extrapolation. Well-designed studies using clinically relevant multispecies models, standardized formulations, and randomized clinical trials are required to establish safety, efficacy, and optimal clinical integration. Until such data are available, electrically charged lipid nanoparticles should be considered experimental adjuncts rather than replacements for established endodontic disinfection protocols.

## Figures and Tables

**Table 1:** Level of clinical evidence of primary experimental studies on electrically charged lipid nanoparticles in endodontics.

Study Type	Number of Studies	Evidence Level
In vitro	10	Preclinical
Ex vivo	4	Preclinical
Animal studies	1	Limited
Clinical observational studies	0	Not available
Randomized clinical trials	0	Not available

**Table 2:** Structural categories of cationic variants of electrically charged lipid nanoparticles.

Nanocarrier Type	Key Features	Reference
**Cationic Liposomes**	Composed of one or more phospholipid bilayers enclosing an aqueous core, suitable for both hydrophilic and lipophilic drugs	Vasiliu et al., 2021 [41]
**Solid Lipid Nanoparticles (SLNs)**	Composed of solid lipids that remain solid at body temperature, offering improved drug retention	Tenchov et al., 2023 [30]
**Nanostructured Lipid Carriers (NLCs)**	Incorporate both solid and liquid lipids to enhance stability and prevent drug expulsion during storage	Marica et al., 2022 [29]

**Table 3:** Electrically charged lipid-based nanocarriers and related systems evaluated in endodontic models.

Type	Composition	Reported (mV)	Particle Size (nm)	Active Agent(s)	Experimental Model	Biofilm/Antibacterial Effect	Reference
Cationic liposomes and BioRoot RCS	NR (liposomal carrying CHX; incorporated into BioRoot RCS bioceramic sealer)	NR	NR	Chlorhexidine digluconate (CHX)	In vitro (sealer + liposomes; antibacterial and release tests)	Complete inhibition of *E. faecalis* biofilm growth versus CHX solution	Raddi et al., 2024 [49]
Liposomes/Invasomes (mTHPC, aPDT)	Liposomes and invasomes with mTHPC (temoporfin)	NR	NR	mTHPC (photosensitizer)	Ex vivo	Up to 3.6-log reduction at canal wall; suppression to 300 µm depth in tubules	Ossmann et al., 2015 [50]
Cationic nanoemulsion (irrigant)	Oil-in-water CHX·HCl nanoemulsion (2% Labrafil, 12% Tween-80, 6% propylene glycol)	NR	12.18	CHX·HCl	In vitro and ex vivo (human teeth; irrigant with/without activation)	Complete eradication at higher CHX% (1.6%); better penetration and cleansing vs. non-nano CHX; PUI most effective activation	Abdelmonem et al., 2019 [51]
Cationic cubosomes (cubosomal CHX)	Cubosomes with CHX (composition per formulation protocol)	+19	157.1 ± 1.2	CHX	Ex vivo (extracted human teeth)	Improved antibiofilm efficacy vs. CHX solution	Mukthamath et al., 2024 [52]
Liposomal CHX (HSPC/Chol)	HSPC/Cholesterol liposomes with CHX	+24.1 to +37.8	148–224	CHX	Ex vivo (extracted human teeth)	Demonstrated deeper penetration and antibacterial effect vs. 2% CHX solution	Shirur et al., 2022 [53]

NR—not reported.

**Table 4:** Comparative Features of Conventional Disinfectants and Nanocarrier-Based Experimental Strategies (Contextual Benchmarking).

Therapy	Biofilm Penetration	Controlled Release	Toxicity Risk	Targeted Delivery	Mechanism of Action	Limitations/Challenges	Evidence Type	Clinical Status	References
Systemic Antibiotics *	Low	No	Moderate	No	Inhibit bacterial metabolism and protein synthesis	Poor penetration into dentinal tubules; systemic side effects; resistance risk	Clinical/In vivo	Standard	Orozco et al., 2025 [65]
NaOCl (Sodium hypochlorite)	Moderate	No	High	No	Oxidation, tissue dissolution, broad bactericidal action	Cytotoxicity to periapical tissues; unpleasant taste/odor; incomplete biofilm removal	In vitro/Ex vivo	Standard	Coaguila-Llerena et al., 2023 [66]
CHX (Chlorhexidine)	Low	No (substantivity only)	Low–Moderate	No	Membrane disruption; residual substantivity	Reduced penetration; staining; less effective on mature biofilms	In vitro/Ex vivo	Standard	Raddi et al., 2024; Shirur et al., 2022 [49,53]
Lipid Nanoparticles (SLNs/NLCs, Liposomes)	High	Yes	Low	Yes	Encapsulation of drugs, electrostatic adhesion to biofilms, sustained release	Mostly preclinical; formulation stability; regulatory approval pending	In vitro/Ex vivo	Preclinical/Experimental	Marica et al., 2022; Ferreira et al., 2025; Ahsan et al., 2024 [29,40,46]
Silver Nanoparticles (AgNPs)	High	Partial	Moderate	Yes	Ag^+^ release, ROS generation, membrane disruption, DNA binding	Potential cytotoxicity at high doses; discoloration; lack of clinical trials	In vitro/Ex vivo	Experimental/Limited FDA-approval in some dental products	Olivares-Acosta et al., 2024 [36]
Polymeric Nanoparticles (e.g., doxycycline-NPs)	Moderate–High	Yes	Variable	Yes	Functionalized for drug loading; gradual release; antibiofilm penetration	Possible immune response; polymer stability	In vitro/Ex vivo	Experimental	Arias-Moliz et al., 2020 [35]
Nanoemulsions (e.g., ciprofloxacin, R-limonene, ZnPc)	Moderate–High	Yes	Low	Yes	Improved solubility, stability, penetration, photodynamic enhancement in some	Efficacy may drop in dentin infection models; require stability optimization	In vitro/Ex vivo	Experimental	Singh et al., 2023; Seker et al., 2022; Schuenck-Rodrigues et al., 2020 [67,68,69]
Graphene-based Nanocarriers (MB-rGO)	High	Yes (with PDT)	Low–Moderate	Yes	Light-activated ROS; graphene improves carrier efficiency	Needs laser activation; light penetration limitations; tooth staining risk	In vitro	Experimental	Jouhar et al., 2025 [70]
Chitosan-based Nanoparticles	High	Yes	Low	Yes	Electrostatic adhesion; drug carrier; EPS disruption	Formulation variability; scaling challenges	In vitro/Ex vivo	Experimental	Abidin et al., 2022; Halkai et al., 2023 [37,71]
Selenium Nanoparticles (SeNPs + PDT)	High	Yes	Low	Yes	ROS generation with PDT; enhanced methylene blue activity	Requires PDT activation; limited in vivo studies	In vitro/Ex vivo	Experimental	Shahmoradi et al., 2021 [72]
Zinc Oxide Nanoparticles (nZOE sealers)	Moderate	Partial	Low–Moderate	Limited	Surface reactivity; ROS generation; membrane penetration	Possible solubility issues; interaction with dentin proteins	In vitro	Experimental	Ghaffari et al., 2024 [73]
Upconversion Nanoparticles (UCNPs, QCh-MB)	High	Yes	Low–Moderate	Yes	Positive surface charge promotes adhesion; NIR-light triggered ROS	Needs NIR laser source; still preclinical	In vitro/Ex vivo	Experimental	Zong et al., 2022 [74]

* Systemic antibiotics are included for mechanistic comparison only and are not recommended for routine root canal disinfection according to AAE/ESE guidelines.

**Table 5:** Cationic nanocarriers evaluated for antibiofilm activity in endodontics (comparative context).

Nanoparticle Type	Composition	Size (nm)	Zeta Potential	Active Agent	Experimental Model	Biofilm Effect	Citations
SLNs/NLCs	Stearic acid or glyceryl monostearate as solid lipid + oleic acid (liquid lipid) + cationic surfactants (CTAB/DDAB)	~100–180 nm	+25 to +40 mV	Photosensitizers (methylene blue, curcumin)	In vitro	Strong reduction in resistant biofilms	Nair et al., 2018 [79]
Cationic solid lipid nanoparticles	Solid lipids (stearic acid, glyceryl monostearate) and cationic surfactant (cetyltrimethylammonium bromide- CTAB)	180–250 nm	+30 to +40 mV	Chlorhexidine	In vitro	Significant reduction in *E. faecalis* biofilm viability (dose-dependent disruption)	Ong et al., 2017 [80]
Chitosan nanoparticles (CSNPs)	Chitosan, cross-linker (TTP, sodium tripolyphosphate) in ionic gelation	~150–200 nm	+28 to +36 mV	Ciprofloxacin, moxifloxacin	In vitro	Significant disruption and elimination of *E. faecalis* biofilms	Pandey et al., 2024 [81]
Liposomes/polymeric nanocarriers	Cationic phospholipids (DOTAP, DODAB), cholesterol	~120–160 nm	+20 to +30 mV	Chlorhexidine, antimicrobial peptides (nisin, LL-37)	In vitro	Improved penetration and effective killing of deep dentinal biofilm compared to free drug; marked decrease in CFU (colony-forming units)	Wang et al., 2020 [82]
DDA-NLC	Nanostructured Lipid Carrier with dimethyldioctadecylammonium bromide	150–250	+20 to +35 mV	Antibiotics/antiseptics	In vitro	Improved penetration and sustained release	Zhang et al., 2010 [8]
DDAB liposomes	Cationic liposome with DDAB	150–250	+20 to +40 mV	Photosensitizers, antibiotics	In vitro/in vivo	Photodynamic biofilm eradication	Leng et al., 2020 [48]
PLGA-methylene blue	Cationic polylactic-co-glycolic (PLGA) nanoparticles loaded with methylene blue	50–100	+25 to +30 mV	Methylene blue	In vitro	Improved penetration, enhanced phototoxicity, greater efficacy than free methylene blue	Kishen et al., 2012 [83]
Ag–NH_2_	Silver nanoparticle with amine functionalization	20–50	+20 to +35 mV	Intrinsic antimicrobial (Ag^+^ release)	In vitro	Potent biofilm kills, low doses effective	Leng et al., 2020 [48]
CTAB-NE	Nanoemulsion with cetyltrimethylammonium bromide	50–150	+25 to +40 mV	Essential oils, antiseptics	In vitro	Rapid membrane disruption, biofilm clearance	Gupta et al., 2016 [84]
BAC-NE	Nanoemulsion with benzalkonium chloride	80–200	+20 to +35 mV	BAC + adjuvants	In vitro	Strong antimicrobial, potential dentin substantivity	Shubhra et al., 2014 [85]
MB-liposomes	Cationic liposomes with methylene blue	100–200	+20 to +35 mV	Methylene blue (photosensitizer)	In vitro/ex vivo	Biofilm eradication with aPDT	Pagonis et al., 2010 [78]
MB-chitosan NP	Chitosan-based nanoparticle with methylene blue	150–250	+25 to +40 mV	Methylene blue	In vitro	Photodynamic killing of *E. faecalis* biofilm	Del Carpio-Perochena et al., 2015 [86]

Non-lipid nanocarriers are included for mechanistic comparison and were not counted among the included studies.

**Table 6:** Summary of Experimental and Review on Nanoparticles in Endodontic Therapy, with emphasis on Lipid-Based Carriers.

Authors	Title	Purpose of the Study	Method	Type of NP	Relevance to the Paper	Main Results
Ferreira et al. * (2025) [40]	Curcumin-Loaded Lipid Nanoparticles.	Evaluation of the antimicrobial efficacy of curcumin through lipid nanoparticles	In vitro experiments against *E. faecalis*	SLN	Use of lipid nanoparticles in dental treatments against bacteria	Curcumin-containing nanoparticles showed high effect against *E. faecalis*, suggesting potential use in endodontic infections.
Ahsan et al. (2024) [14]	Lipid Nanocarriers-Enabled Delivery of Antibiotics…	Improving Antibiotic Penetration in Biofilms via Lipid Carriers	Theoretical Summary and Preliminary Studies	SLNs, NLCs, liposomes	Adds context to the efficacy of antibiotics in biofilms	Delivery through lipid carriers outweighs the resistance of biofilms and increases the local concentration of antibiotics.
Diogo et al. (2023) [77]	May carriers at nanoscale improve the Endodontic’s future?	Research on the role of nanoscale carriers in endodontics	Summary article	Liposomes, niosomes, transferosomes	Elaborates on the importance of nanotechnology in endodontics	Nanocarriers have great potential for controlled delivery and improved treatment in endodontics.
Lee et al. (2022) [44]	Lipid-coated hybrid nanoparticles for enhanced bacterial biofilm penetration…	Testing Hybrid Nanoparticles to Penetrate Bacterial Biofilms	Laboratory study	Hybrid nanoparticles	Focus on the penetration and effect of electric charge	Hybrid nanoparticles penetrated deeper into biofilms and showed high antibiofilm efficacy.
Panthi et al. (2024) [87]	Liposomal drug delivery strategies…	Liposome Strategies to Combat Bacterial Biofilms	Summary article with experimental data	liposomes	Reinforces the concept of targeted antibiotic delivery	Liposomes were achieved to improve the penetration and action of antibiotics against bacterial biofilms.
Forier et al. (2014) [88]	Lipid and polymer nanoparticles for drug delivery to bacterial biofilms	Analysis of the Use of Nanoparticles for Biofilms	Literature Summary	Lipid and polymeric nanoparticles	Adds the scientific dimension of target delivery	Lipid and polymeric nanoparticles offered better efficiency in delivering drugs to biofilms.
Hou et al. (2021) [43]	Lipid nanoparticles for mRNA delivery	Lipid Nanoparticle Technology Explained	Review: background/engineering	Lipid nanoparticles (mRNA delivery, engineering background)	Support the theoretical construction of the delivery system	The lipid nanoparticles for mRNA were safe and effective in delivery, showing potential to be used for other medical applications.
Tenchov et al. (2021) [30]	Lipid nanoparticles—from liposomes to mRNA vaccine delivery…	Description of the evolution of lipid nanoparticles	Summary article: background/engineering	Lipid nanoparticles (general, engineering background)	Support for the technological importance of the method	Lipid nanoparticle technologies have become a mainstay in modern therapies, including antibacterial ones.
Liu et al. (2023) [89]	Glucose-Responsive Charge-Switchable lipid nanoparticles…	Intelligent delivery via nanoparticles with controllable charge	Experimental study: background/engineering	Charge-switchable lipid nanoparticles (biomedical engineering)	Supports the electric charge and discharge control part	The ‘charge-switchable’ nanoparticles offered precise control of drug release and response to the environment (glucose).
Xie et al. (2025) [15]	Nanomaterial-enabled anti-biofilm strategies…	Innovation in the use of nanomaterials to combat biofilm	Summary of recent advancements	Multiple types of NPs	Relies on modern challenges and solutions for biofilms	Strategies with nanomaterials significantly increased efficacy against biofilm without damaging the surrounding tissue.

* This article focuses on electrically charged solid lipid nanoparticles (SLNs). Non-SLN nanocarriers (liposomes, polymeric or hybrid nanoparticles) are included in the table for mechanistic comparison as they provide insights into charge effects, biofilm penetration, and controlled release relevant to SLN design.

## Data Availability

The original contributions presented in this study are included in the article and Supplementary Materials. Further inquiries can be directed to the corresponding authors.

## Supplementary Materials

The following supporting information can be downloaded at: https://www.mdpi.com/article/10.3390/dj14030171/s1. Table S1: PRISMA_2020_checklist; File S1: Search strategies (long version with each database; File S2: Full-text articles excluded after eligibility assessment (n = 12).
