# Supplementary material for: Electrically Charged Lipid Nanoparticles as Intracanal Antimicrobial Delivery Systems: A Narrative Review of Preclinical Evidence for Biofilm Control
Source: Dent J (Basel). 2026 Mar 16;14(3):171. doi: 10.3390/dj14030171 (PMC13024947; doi:10.3390/dj14030171)
Supplement: Supplementary file 1 [file dentistry-14-00171-s001.zip › File S1 Search strategies long version with each database.pdf]

Supplementary Appendix S1. Search strategies (long version with each database)

Databases searched

PubMed (MEDLINE), Scopus, and Web of Science Core Collection.

Time window and date last searched

- Time window applied: January 2010 – December 2025
- Final search date: 1 January 2026

Records retrieved

The combined export total prior to deduplication was 312 records (PubMed + Scopus + Web of Science). Per-database record counts were not retained.

1) PubMed (MEDLINE)

Search string (as executed)

(endodont* OR "root canal" OR dentin OR endodontic)
AND
(nanoparticle* OR "lipid nanoparticle*" OR liposome* OR nanoemulsion* OR "solid lipid" OR "nanostructured lipid" OR chitosan OR "polyethyleneimine" OR PEI OR "quatern* chitosan" OR "cationic polymer*" OR "zinc oxide" OR ZnO)
AND
(cationic OR "positively charged" OR "surface charge" OR zeta)
AND
(biofilm OR antibacter* OR antimicrobial OR antibiotic OR "drug delivery" OR irrigant OR medicament)
AND
("in vitro" OR "ex vivo" OR "in vivo" OR animal OR rat OR canine OR dog OR rodent)

Filters/limits applied

- Publication dates: 2010/01/01 – 2025/12/31
- Language: English
- Article type: Journal Article
- Excluded: Reviews, Editorials, Letters

2) Scopus

Search field

TITLE-ABS-KEY

Search string (as executed/adapted to database syntax)

TITLE-ABS-KEY (endodont* OR "root canal" OR dentin OR endodontic)
AND TITLE-ABS-KEY (nanoparticle* OR "lipid nanoparticle*" OR liposome* OR nanoemulsion* OR "solid lipid" OR "nanostructured lipid" OR chitosan OR "polyethyleneimine" OR PEI OR "quatern* chitosan" OR "cationic polymer*" OR "zinc oxide" OR ZnO)
AND TITLE-ABS-KEY (cationic OR "positively charged" OR "surface charge" OR zeta)
AND TITLE-ABS-KEY (biofilm OR antibacter* OR antimicrobial OR antibiotic OR "drug delivery" OR irrigant OR medicament)
AND TITLE-ABS-KEY ("in vitro" OR "ex vivo" OR "in vivo" OR animal OR rat OR canine OR dog OR rodent)

Filters/limits applied

- Year: 2010–2025
- Document type: Article
- Language: English
- Subject areas (as applied): Dentistry; Materials Science; Medicine

3) Web of Science Core Collection

Search field

TOPIC (TS)

Search string (as executed/adapted to database syntax)

(endodont* OR "root canal" OR dentin OR endodontic)
AND
(nanoparticle* OR "lipid nanoparticle*" OR liposome* OR nanoemulsion* OR "solid lipid" OR "nanostructured lipid" OR chitosan OR "polyethyleneimine" OR PEI OR "quatern* chitosan" OR "cationic polymer*" OR "zinc oxide" OR ZnO)
AND
(cationic OR "positively charged" OR "surface charge" OR zeta)
AND

(biofilm OR antibacter* OR antimicrobial OR antibiotic OR "drug delivery" OR irrigant OR medicament)
AND
("in vitro" OR "ex vivo" OR "in vivo" OR animal OR rat OR canine OR dog OR rodent)

Filters/limits applied

- Timespan: 2010–2025
- Document type: Article
- Language: English
- Categories (as applied): Dentistry Oral Surgery Medicine; Materials Science; Nanoscience Nanotechnology

Additional searching

Reference lists of relevant review articles were screened to identify eligible studies not captured in the electronic search.
