# Supplementary material for: Electrically Charged Lipid Nanoparticles as Intracanal Antimicrobial Delivery Systems: A Narrative Review of Preclinical Evidence for Biofilm Control
Source: Dent J (Basel). 2026 Mar 16;14(3):171. doi: 10.3390/dj14030171 (PMC13024947; doi:10.3390/dj14030171)
Supplement: Supplementary file 1 [file dentistry-14-00171-s001.zip › File S2 Full text articles excluded after eligibility assessment n=12.pdf]

Full-text exclusion breakdown:

File S2. Full-text articles excluded after eligibility assessment (n = 12)

Each article was assigned a single primary exclusion reason according to predefined eligibility criteria.

A) Non-lipid nanoparticle formulations (n = 5)

Study (first author, year)	Reason for exclusion
**Kishen, 2008 [83]**	Non-lipid nanoparticles (zinc oxide/chitosan); outside lipid nanoparticle scope
**Del Carpio-Perochena, 2022 [86]**	Chitosan-based nanocomplex modification (non-lipid system)
**Arias-Moliz, 2020 [35]**	Polymeric nanoparticles; not lipid nanoparticles/liposomes
**Ertem, 2017**	Core–shell silver nanoparticles (AgNPs@SiO_2); inorganic nanoparticles
**Pushpalatha, 2022**	Zinc oxide nanoparticles (metal-oxide); non-lipid system

B) Lack of relevance to endodontic disinfection (n = 4)

Study (first author, year)	Reason for exclusion
**Léber, 2018**	Periodontal drug-delivery system; not root-canal disinfection
**Salar, 2019**	Insulin encapsulation/drug delivery; not endodontic application
**Müller, 2000**	General SLN drug-delivery review; not endodontic disinfection
**Foroughi, 2025**	Irrigant/activation study without lipid nanoparticle intervention

C) Insufficient quantitative or semi-quantitative outcome data for extraction (n = 3)

Study (first author, year)	Reason for exclusion
**Chandak, 2021**	Narrative review; no extractable primary outcome data
**Ivković, 2025 [33]**	Scoping review; no extractable quantitative antimicrobial data
**RSC Advances review, 2020**	Broad nanomaterials dentistry review; no extractable endodontic disinfection outcomes
